# A Novel Domain Transfer-Based Approach for Unsupervised Thermal Image Super-Resolution

**DOI:** 10.3390/s22062254

**Published:** 2022-03-14

**Authors:** Rafael E. Rivadeneira, Angel D. Sappa, Boris X. Vintimilla, Riad Hammoud

**Affiliations:** 1 Escuela Superior Politécnica del Litoral, ESPOL, Facultad de Ingeniería en Electricidad y Computación, CIDIS, Campus Gustavo Galindo Km. 30.5 Vía Perimetral, P.O. Box 09-01-5863, Guayaquil 090112, Ecuador or asappa@cvc.uab.es (A.D.S.); boris.vintimilla@espol.edu.ec (B.X.V.); 2 Computer Vision Center, Edifici O, Campus UAB, Bellaterra, 08193 Barcelona, Spain; 3 TuSimple Inc., 9191 Towne Centre Dr. Ste 600, San Diego, CA 92122, USA; hammoud@csail.mit.edu

**Keywords:** thermal image super-resolution, unsupervised super-resolution, thermal images, attention module, semiregistered thermal images

## Abstract

This paper presents a transfer domain strategy to tackle the limitations of low-resolution thermal sensors and generate higher-resolution images of reasonable quality. The proposed technique employs a CycleGAN architecture and uses a ResNet as an encoder in the generator along with an attention module and a novel loss function. The network is trained on a multi-resolution thermal image dataset acquired with three different thermal sensors. Results report better performance benchmarking results on the 2nd CVPR-PBVS-2021 thermal image super-resolution challenge than state-of-the-art methods. The code of this work is available online.

## 1. Introduction

Single image super-resolution (SISR) is a classical computer vision problem that tries to infer a high-resolution (HR) image from a single low-resolution (LR) input image. This problem is still an active research field in the computer vision community (e.g., [1,2,3,4]). Several applications in different fields can benefit from super-resolution (SR) representations, for instance, security (e.g., [5,6]), medical imaging (e.g., [7]), object detection (e.g., [8]), and astronomical images (e.g., [9]), among others. Different methods have been proposed to deal with the SISR problem; most of them are tackled with machine learning (ML) techniques and deep convolutional neural networks (CNN) methods. CNN-based methods for SISR can learn the mapping function from LR to HR image. Concerning state-of-the-art approaches, most of them have been intended to tackle the SR problem in visible spectrum domain—i.e., RGB images, using deep learning techniques by directly training networks to capture the LR to HR mapping.

In recent years, long-wavelength infrared (LWIR) images, a.k.a. thermal images, have shown to be useful to efficiently solve problems from different domains (e.g., security monitor [10], medical imaging [11], car assistance [12], visual inspection [13], and human detection [14], among others) because thermal images have the information of the radiation emitted by the surface of an object (temperature above zero [15]) captured by thermal cameras. As mentioned above, thermal cameras play an important role in different areas. Unfortunately, most affordable thermal cameras have poor resolution, and high-resolution ones are still expensive nowadays. A possible way to overcome this limitation could be to develop a CNN-based architecture to generate an HR representation from a given LR image. In actuality, in recent years, single thermal image super-resolution has become an active research topic in the computer vision community.

In order to develop new CNN architectures and train them, it is crucial to have a large HR dataset. In the visible spectrum, thousands of HR images can be used for such a task; unfortunately, in the thermal image domain, most of the available datasets tend to have a poor resolution or do not present a high variability needed to generalize the training. Due to this lack of thermal images, a novel dataset was proposed in [16] with three different resolutions (low, mid, and high) obtained with three different thermal cameras. This dataset has been used as a benchmark in the first and second thermal image super-resolution challenge on PBVS-CVPR2020 [17], and PBVS-CVPR2021 (http://pbvs-workshop.github.io) (accessed on 12 December 2021) [18] workshops, where several teams around the world have participated, and a baseline has been obtained.

Keeping in mind the limitation mentioned above of lack of large thermal image datasets, a novel CycleGAN architecture is proposed in the current work. It is based on the usage of a novel loss function (SOBEL cycle loss) together with an attention module (AM) in the bottleneck of the generator. This unsupervised approach achieves results better than those obtained in the second evaluation of the PBVS-CVPR2021 challenge. This approach takes into consideration the gap between the generated and real HR images.

In summary, the main contributions of this manuscript are as follows:

- Improve results from previous work by using a CycleGAN-based approach with novel losses functions.
- Use an attention module in the generator for a better high feature extraction reaching better results.
- Evaluate the approach with different datasets overcoming state-of-the-art results.

The remainder of this paper is organized as follows: Section 2 presents works related to the topics of the current work. The proposed architecture is detailed in Section 3. Results are provided in Section 4. Finally, conclusions are given in Section 5.

## 2. Related Work

As mentioned above, most of the single image super-resolution works are focused on the visible spectrum. Hence, despite the fact this work is focused on the thermal image super-resolution, this section reviews the most representative visible spectrum state-of-the-art SISR approaches and other spectral bands such as near- and far-infrared SR approaches. This section starts by reviewing the most typical thermal image datasets used as benchmarks by the research community.

### 2.1. Benchmark Datasets

In visible spectrum, there is a large number of HR datasets available for training and evaluating the performance of SR networks (e.g., [19,20,21,22,23,24], among others). The acquisition of these HR images in different scenarios with a large set of objects’ categories (e.g., people, building, animals, clothes, food, and cars, among others) is easy to obtain since visible spectrum cameras are widely available. On the contrary, in the thermal image domain, there are just a few datasets available (e.g., [25,26,27], among others), most of them in low resolution or from the same scenario (low variability). Other thermal images datasets were acquired for other specific applications (e.g., biometric domain, medical, security) but used to tackle the thermal image super-resolution problem. As far as we know, [28] has the largest HR thermal images dataset available in the literature, collected with an FLIR SC8000 in a full-resolution of 1024 × 1024 pixels. The main drawback with this dataset is that all these images are from the same scenario.

As a contribution to overcoming the lack of thermal image datasets intended for the SR tasks, [29] presents a novel dataset. It has 101 HR thermal images acquired with a TAU2 FLIR camera, in a native resolution of 640 × 512 pixels of different scenarios (e.g., indoor, outdoor, day, night, objects). In addition, a large dataset was released by FLIR company FREE FLIR Thermal Dataset for Algorithm Training (https://www.flir.in/oem/adas/adas-dataset-form/) (accessed on 12 December 2021), focused on training and validation object detection. This dataset was acquired with a TAU2 mounted on a vehicle providing a total of 14,452 thermal images, with a 640 × 512 resolution. This dataset was intended for driving assistance applications, although it can be used for the super-resolution problem.

The datasets mentioned above contain images obtained from one thermal camera, and most of these datasets are not large enough. To reach good results in CNN architectures’ training and evaluation processes, it is essential to have a large dataset. Recently, [16] presents a novel dataset that consists of a set of 1021 thermal images acquired with three different thermal cameras at different resolutions. This dataset contains different outdoor scenarios (e.g., morning, afternoon, and night) and objects (e.g., people, cars, buildings, vegetation). The cameras were mounted on a panel, trying to minimize the baseline distance between the optical axis to obtain an almost registered image set. This dataset was used as a benchmark in the first and second thermal image super-resolution challenge organized on the workshop *Perception Beyond the Visible Spectrum* of CVPR2020 [17] and CVPR2021 conferences [18].

### 2.2. Super-Resolution Approaches

Image super-resolution is a classical issue studied in the literature for years and is still a challenging problem in the computer vision community. It can be categorized as single-image SR (SISR) and multi-image SR (MISR). SISR is more challenging than MISR due to the lack of features that can be extracted in just one image rather than multiple images of the same scene. Due to the large amount of literature in the visible spectrum, this section starts first by reviewing approaches intended for visible spectrum images and then approaches for thermal images are reviewed.

Nowadays, the uses of CNN-based methods are the mainstream in SISR. Deep learning-based SISR techniques were firstly introduced by [30] in 2015, proposing a simple three-layer convolutional neural network called SRCNN. It aims to learn a direct mapping between low- and high-resolution image pairs, showing the capability to improve the quality of SR results compared to traditional methods (e.g., bicubic interpolation). After SRCNN, several network architectures were proposed. [31] presents an approach called VDSR which makes use of global residual learning and increasing the depth of the network from 3 to 20 layers. For a better computational performance, [32] proposes an architecture called FSRCNN; this architecture extracts the feature maps on the low-resolution image and inserts a deconvolution layer for SR reconstruction, which learns an end-to-end mapping. In recent years, different approaches have been published using deeper networks (e.g., [31,33,34]) with more convolutional layers and residual learning or densely connected networks. Unfortunately, these deep architectures consume a lot of computational resources.

The CNNs mentioned above have been proposed for visible spectrum images and aim to minimize the difference between SR and HR images using a supervised training process. This process has to have a pixelwise registration between LR and HR (pair of images), and, usually, these approaches downsample the given HR image, add random blur or noise on it, and then use it as the input LR image. Unfortunately, the fixed degradation assumption limits their performances when real low-resolution images need to be processed.

Recently, unsupervised super-resolution approaches have been proposed to leverage unpaired images to overcome the limitation of having a pixelwise registration without any assumption on the degradation model. Refs. [35,36] propose to use an adversarial objective function that uses multitask loss formulation. Some unsupervised training processes have been presented, such as image colorization [37], transferring style [38], feature estimation [39], and image enhancement [40], among others. These approaches are based on two-way GANs networks (a.k.a. CycleGAN) that can learn from an unpaired set of images [41], widely used in image-to-image translation. With CycleGAN architectures, it is possible to map images from one domain into another domain. When there is not a pixelwise registration or in the absence of paired examples, CycleGAN functionality makes models appropriate for image SR estimation.

Attention mechanisms are present in novel architectures, becoming an integral part of models. Some models (e.g., [42,43,44,45], among others) have shown that the use of attention mechanism improves the performance and visual effect, because the attention network allocates attention away from the noisy channels. A self-attention of the GAN framework is introduced in [46], enabling the generator and the discriminator to model the relationships between widely separated spatial regions.

Most of the SR approaches mentioned above are focused on images from the visible spectrum. Super-resolution strategies have also been proposed to enhance the resolution of images from other spectral bands. For near-infrared spectrum, [47] proposes a novel image SR method via discriminative dictionary and deep residual network. For hyperspectral image SR, [48] proposes a fast low tensor multi-rank that speeds up the estimation of spectral coefficient and preserves the prior information of hyperspectral images. Inspired in SRCNN, [49] proposes the first approach for thermal image super-resolution, called TEN, where the authors train the network using RGB images due the lack of thermal images. In [50], the authors use the luminance channels (by transforming images from RGB to YCbCr color space) and train the network with the Y channel; then, the network parameters are fine-tuned with thermal images. A denoising method is proposed in [43] to solve poor image quality and noise removal on thermal imaging based on a second-order channel attention mechanism. In addition, [51] proposes a novel SR and deblurring method using a GAN architecture for thermal images. In [29], they conclude that better results are obtained if the network is trained using images from the same spectral band. Additionally, [16] trains a CycleGAN architecture for transfer of an LR image domain (from one camera) to an HR image domain (of another camera) without having registered pairs of images.

An important factor consider is the criteria used for evaluation; in the first thermal image super-resolution challenge [17], using the dataset from [29], two kinds of evaluations are proposed. Evaluation 1 consists of downsampling the HR thermal images by ×2, ×3, and ×4 and comparing their SR results with the corresponding GT images. Evaluation 2 consists of obtaining the ×2 SR from a given MR thermal image and comparing it with its corresponding semiregistered HR image. Several teams have participated in this challenge and presented their approaches improving the results of peak signal-to-noise ratio (PSNR) and Structural Similarity Index Measure (SSIM) metrics benchmark. The best results according to the evaluations mention above were MLVC-Lab [52] and Couger AI [53] architectures, winner of Evaluation 1 and Evaluation 2, respectively. MLVC-Lab team presents a new ResBlock module, which uses local and long skip connections where the higher layer gradients are bypassed to the lower layer, avoiding the higher layer gradients directly to the first convolution layer. A channel attention module is adopted to rescale the channel-wise features. The Couger AI team proposes an architecture based on a neural network that uses coordinate convolutional layer and residual units, along with the multilevel supervision and attention unit to map the information between LR to MR and HR images.

At the second thermal image super-resolution challenge [18], different teams also participated and presented their approaches. For this second challenge, the same dataset is used, but for Evaluation 1, just ×4 on HR were considered and Evaluation 2 maintains the same method (MR to HR images). Taking into consideration just the results of Evaluation 2, the present work is compared with the three best results of the challenge, which are from the ULB-LISA, SVNIT-NTNU-2, and NPU-MPI-LAB teams. The ULB-LISA team introduces a model referred to as the xcycles backprojection network (XCBP), composed of a cycle features correction (CFC) and residual features extraction (RFE). The SVNIT-NTNU-2 team [54] employs a GAN framework for semisupervised learning using a UNet-based network. NPU-MPI-LAB uses a network inspired by ESRGAN to deal with SR and domain adaptation at the same time.

The two challenges mentioned above show the interest of the active community in the thermal image SR. The results from these challenges are clear examples of how architectures are evolving, improving the results on the different metrics. These results can be used as a baseline for future works in the community.

## 3. Proposed Approach

As mentioned above, the current work is based on the usage of a CycleGAN architecture intended to overcome the lack of large thermal image datasets. Since no pairwise data are required, a larger set of training data is considered by using an LR image from one camera together with a HR image from another camera. Section 3.1 shows the proposed architecture and loss functions. Then, Section 3.2 presents the datasets used for training and validation. Finally, the strategy used to evaluate the proposed approach is detailed in Section 3.3.

### 3.1. Architecture

**Figure 1:**
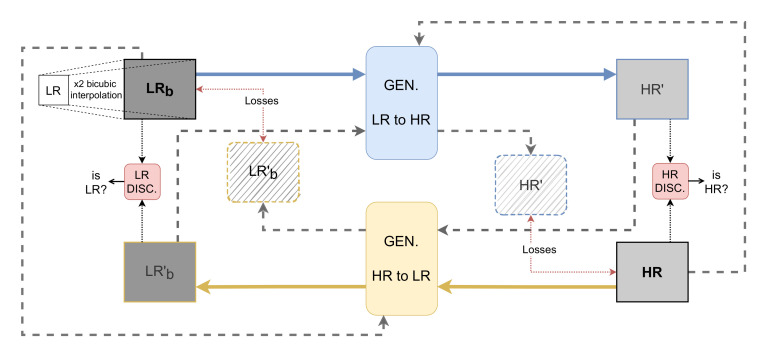
CycleGAN-based architecture with 6 blocks ResNet as a generator (for LR to HR and vice versa); losses represent adversarial, Sobel, cycled, SSIM and identity loss. Each cycle has its respective discriminators.

**Figure 2:**
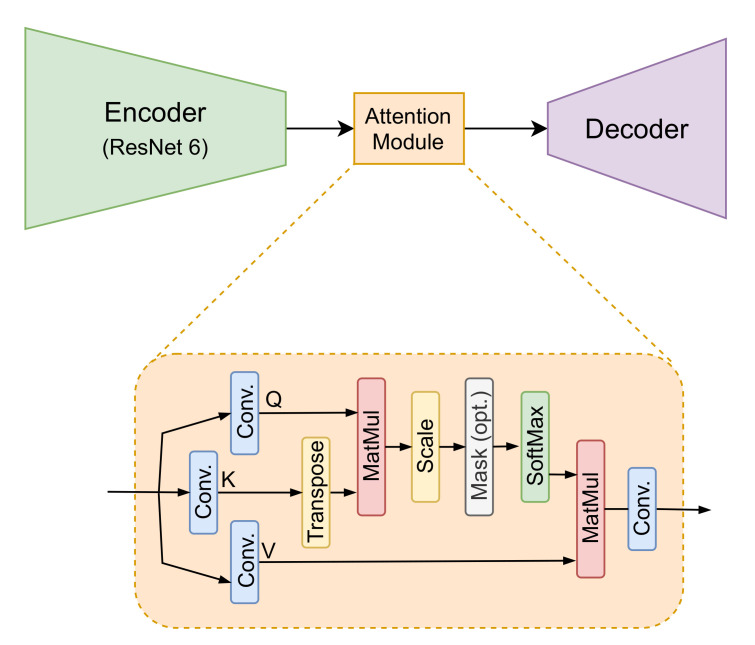
Generator defined by 6 blocks ResNet as encoder, followed by the scaled dot-product attention module [44] and then the decoder.

The cycle generative adversarial network (CycleGAN) [41], widely used for mapping features from one domain to another domain for image-to-image translation tasks in the absence of paired examples images, is used in the current work. This framework is used to learn a mapping from the low-resolution (LR) to the high-resolution (HR) domain solving the SR problem. This is a recursive process where the mapping functions try to generate images with a similar distribution at each domain. The proposed approach, shown in Figure 1, consists of two generators, from LR domain to HR domain and vice versa. Each has its corresponding discriminator that validates the generated images. The generators are a ResNet with six residual blocks (ResNet-6). The residual blocks have convolutional layers, with instance normalization and ReLu activation with skip connections. Inspired in [46], an attention module is added after the ResNet Encoder step (at the bottleneck of the generator), as shown in Figure 2. A patchGAN architecture is considered as a discriminator; for validation, the non-paired GT image and the generated image are used to validate if the output is real or not.

The attention module is a scaled dot-product as proposed in [44], which consists of the operation of three weight matrix, as shown in Figure 2, obtained from a convolution operation of the last output layer in the encoder. The attention output is computed as follows:

(1) $$Attention\left(Q,K,V\right)=softmax\left(\frac{QK^{T}}{\sqrt{d_{k}}}\right)V,$$

where Q, K, and V refer to query, key, and value, respectively. T refers to transpose operation on key matrix. The dot product of the query is computed with all keys, and the softmax function is applied to obtain the weights on the values. They are the input matrices that contain the feature representation of the encoder, and d${}_{k}$ is a scaled-down factor. The scaling is performed so that softmax function’s arguments do not become excessively large with a higher dimension.

Following the architecture presented in [16], a combination of different loss functions is used: i) adversarial loss $L_{Adversarial}$, ii) cycle loss $L_{Cycle}$, iii) identity loss $L_{Identity}$, and iv) structural similarity loss $L_{SSIM}$; additionally, another loss term, Sobel loss $L_{Sobel}$, is proposed. Sobel loss consists of applying Sobel edge detector [55] to the input image and the cycled generated image and obtain the mean square difference between both images; it helps to evaluate the contour consistency between the two images. All these loss function terms are intended to obtain an HR representation with the highest fidelity (i.e., accuracy on temperature information on reconstructed thermal images).

The **adversarial loss** is designed to minimize the cross-entropy to improve the texture loss:

(2) $$L_{Adversarial}=-\sum_{i}logD\left(G_{L2H}\left(I_{L}\right),I_{H}\right),$$

where *D* is the discriminator, $G_{L2H}\left(I_{L}\right)$ is the generated image, and $I_{L}$ and $I_{H}$ are the low- and high-resolution images, respectively.

**Figure 3:**
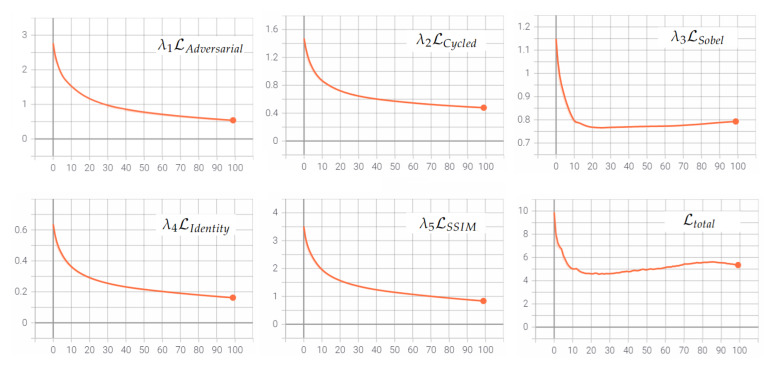
Adversarial, cycled, Sobel, identity, and SSIM losses from generator LR to HR and the total loss.

The **cycled loss** ($L_{Cycled}$) is used to determinate the consistency between input and cycled output; it is defined as

(3) $$L_{Cycled}=\frac{1}{N}\sum_{i}\left|\right|G_{H2L}\left(G_{L2H}\left(I_{L}\right)\right)-I_{L}\left|\right|,$$

where $G_{L2H}$ and $G_{H2L}$ are the generators that go from one domain to the other domain. The **Sobel loss** ($L_{Sobel}$) is used to determinate the edge consistency between input and cycled output; it is defined as

(4) $$L_{Sobel}=\frac{1}{N}\sum_{i}||Sobel\left(G_{H2L}\left(G_{L2H}\left(I_{L}\right)\right)\right)-Sobel\left(I_{L}\right)\left|\right|,$$

where $G_{L2H}$ and $G_{H2L}$ are the generators that go from one domain to the other domain, and Sobel obtains the edges of each of the objects in the images. The **identity loss** ($L_{Identity}$) is used for maintaining the consistency between input and output; it is defined as

(5) $$L_{Identity}=\frac{1}{N}\sum_{i}\left|\right|G_{H2L}\left(I_{L}\right)-I_{L}\left|\right|,$$

where *G* is the generated image and *I* is the input image. The **structural similarity loss** ($L_{SSIM}$) for a pixel *P* is defined as

(6) $$L_{SSIM}=\frac{1}{NM}\sum_{p=1}^{P}1-SSIM\left(p\right),$$

where SSIM(p) is the structural similarity index (see [56] for more details) centered in pixel *p* of the patch (P). The **total loss function** ($L_{total}$) used in this work is the weighted sum of the individual loss function terms:

(7) $$L_{total}=\lambda_{1}L_{Adversarial}+\lambda_{2}L_{Cycled}+\lambda_{3}L_{Sobel}+\lambda_{4}L_{Identity}+\lambda_{5}L_{SSIM},$$

where $\lambda_{i}$ parameters for adversarial, cycled, and identity losses are maintained as the original CycleGAN proposed, and for SSIM and Sobel losses were set empirically according to best results of the experiments; cycled and SSIM losses were set with a higher value. Separate losses vs. epoch plots from LR2HR generator are shown in Figure 3.

### 3.2. Datasets

**Figure 4:**
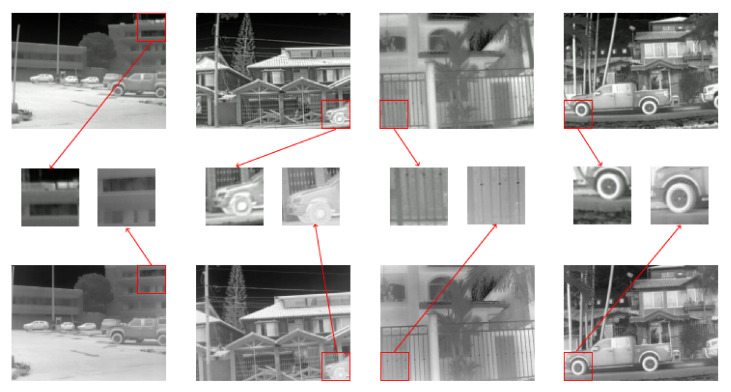
Examples of thermal images from [16]: (**top**) MR images from Axis Q2901-E (320 × 240), used in the current work as LR images; (**bottom**) HR images from FC-6320 FLIR (640 × 480) [16]; (**middle**) enlargements to show the misregistration between the images.

**Figure 5:**
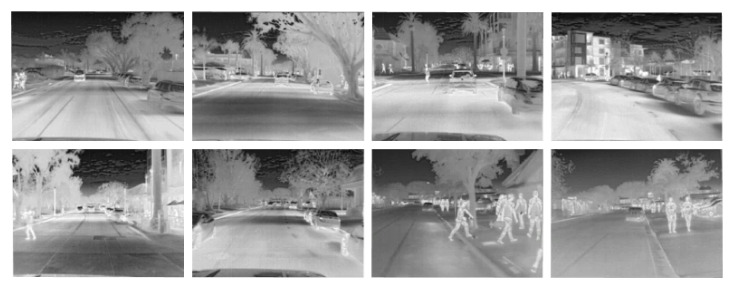
Examples of the *Free FLIR Thermal Dataset for Algorithm Training* (FLIR-ADAS).

Two of the datasets mentioned in Section 2.1 have been considered for training the proposed approach. The first dataset, from [16], has images acquired with three different cameras at different resolutions; each resolution set has 951 images for training, 50 for validation, and 20 images are left for testing. Only mid-resolution images (considered as LR inputs in the current work) and high-resolution images (HR) are considered; Figure 4 shows some illustrations of this dataset. It is worth noticing that the input images (LR and HR) are from different cameras and they are not pixelwise registered. The second dataset used in the current work is a video sequence with 8862 thermal images from *FREE FLIR Thermal Dataset for Algorithm Training*; just 985 images were selected (one out of nine images) to have a more variance scenario, Figure 5 shows some illustrations of this second dataset. HR images from both datasets have a native resolution of 640 × 512; these HR images are centered cropped to 640 × 480 on both datasets, in order to exactly have ×2 size resolution regarding LR images. Both datasets have the same format (8 bits in jpg format) but are acquired in different places and conditions.

### 3.3. Evaluation

**Figure 6:**
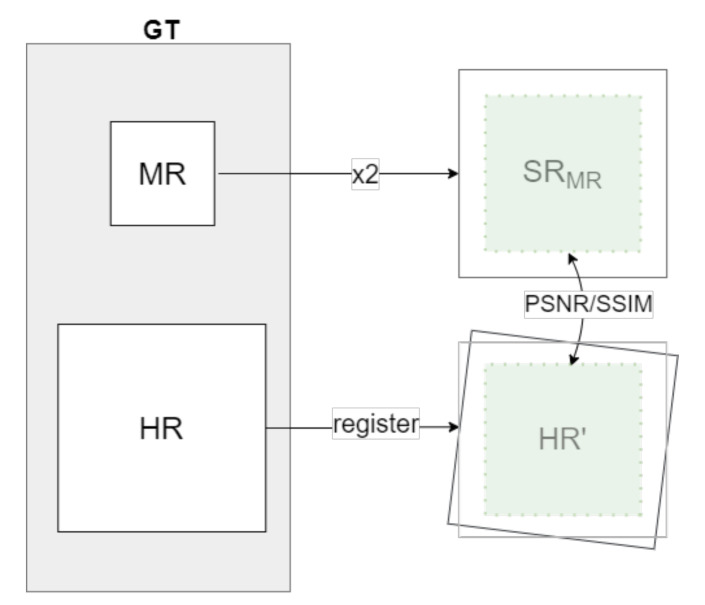
Illustration of the Evaluation 2 criteria from PBVS-CVPR2021 challenge [17] (mid- to high-resolution domain dataset).

As proposed in [16], and adopted in the PBVS-CVPR2021 challenge [18] (referred to as a *Evaluation 2*), the quantitative evaluation of the approach presented in the current work is performed by means of the average PSNR and SSIM measures between the generated SR image and the semiregistered HR counterpart obtained from the other camera; this evaluation is illustrated in Figure 6. Due to the camera baseline, the information in the images is not the same; hence, just an ROI of the 80% of the image size, centered at each image, is considered. For a fair comparison, the same validation set as PBVS-CVPR2021 is used.

## 4. Experimental Results

The results obtained from the proposed unsupervised thermal image super-resolution architecture are depicted in the current section. Section 4.1 describes the settings, while Section 4.2 presents the quantitative results. Additionally, the code of this work has been published, and it is available at https://github.com/rafariva/unsupervisedThSR (accessed on 12 December 2021).

### 4.1. Settings

The proposed architecture was trained in a NVIDIA Titan X mounted in a workstation with 128 GB of RAM. Python programming language and Tensorflow 2.0 library were used. Only the two datasets mentioned in Section 2.1 were considered. No data-augmentation process was applied to the given input data.

CycleGAN transfer domain needs images at the same resolution; hence, the input images (LR) are upsampled by bicubic interpolation and normalized in a [−1, 1] range—note, this normalization is performed to make computation efficient and avoid memory problems during the training process but resulting HR image is represented back in grayscale, in order to obtain temperature values (i.e., white pixels correspond to hot spot, while black pixels to cold spot). The training process is performed for 100 epochs without dropout (the model does not present overfitting). As a generator, a ResNet with six residual blocks (ResNet-6) is used. Stochastic AdamOptimizer is used to prevent overfittings and lead to faster convergence, avoiding degradation during the training. After the encoder phase, as shown in Figure 2, an attention module is set, which performs three separate convolutions to the output of the encoder. As a discriminator, patchGAN architecture is used. It validates if the generated images together with the GT images are real or not. During the training, in each epoch, the input images are randomly selected according to the batch size. The learning rate is set to 0.0002 for both generator and discriminator networks; epsilon = 1 × $10^{-5}$; exponential decay rate for the 1st momentum, 0.5 for the discriminator, and 0.4 for the generator. The $\lambda_{i}$ values that weigh each loss are set as follows: $L_{Cycled}$ = 10, $L_{Identity}$ = 5, $L_{SSIM}$ = 5, and $L_{Sobel}$ = 10 in order to reach the best results, where the cycled and Sobel losses have higher values for the importance in their corresponding loss functions.

The proposed architecture was trained four times, one with just the first dataset and once with both datasets together, then one more for each but with and without the attention module. As the second dataset are frame images from a video sequence, and for having more variability and the images of a balanced number as the first dataset, every nine frames were selected. As mentioned above, in Section 3.3, the validation was performed with the same set of images used in the PBVS-CVPR2021 challenge [18], to compare the current results with the most recent results in the state-of-the-art literature.

### 4.2. Results

**Figure 7:**
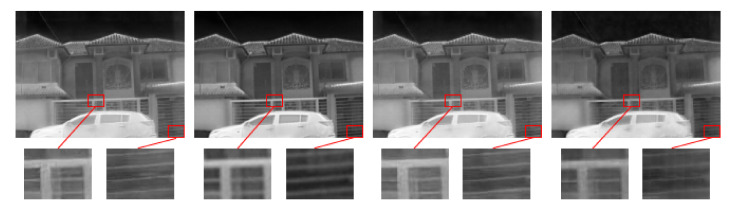
Visual comparison of SR results obtained using work${}^{1}$ (PA-D1), work${}^{2}$ (PA-D1-D2), work${}^{3}$ (PA-D1-AT), and work${}^{4}$ (PA-D1-D2-AT), respectively.

Quantitative results from the proposed architecture are compared with the best three approaches from the PBVS-CVPR2021 challenge. Table 1 depicts PSNR and SSIM measures for the comparisons. The best result is highlighted in bold, and the second-best result is underlined. Qualitative results are depicted in Figure 7.

As can be appreciated, the approach that reaches the best result in PSNR metric (current work${}^{3}$) uses the attention module; it achieves the third-best result in SSIM. This approach was trained with just the first dataset. The approach without attention module but trained with both datasets (current work${}^{2}$) preserves the structural information (SSIM) better than other methods. The usage of just the first dataset shows a good performance; this means that this dataset has a large enough variability to train a network and that it is possible to perform a single thermal image super-resolution between two different domains using images acquired with different camera resolutions and without registration. The used validation and testing images set are from the same D1 dataset (with different kinds of scenarios), meanwhile the D2 dataset is from a video sequence from just street scenario. This causes a bias in the network regarding PSNR measure.

Regarding our previous work, the present approach shows better results by adding and adjusting losses functions variation and, better yet, with the attention module. Using both datasets (without attention module) increases SSIM measure, (reaching the best result in this measure), but with attention module using just the first dataset overcomes the best result in PSNR measurement and better SSIM measurement than previous work. With some changes from previous work, quantitative measures overcome previous results and also are the best approaches from PBVS-CVPR2021 challenge (Evaluation 2).

## 5. Conclusions

This paper presents an improved version of our previous work [16]. Two datasets are considered during the training stage with different hyperparameters values adjustment. The proposed CycleGAN architecture uses a Sobel loss and an attention module, in between the encoder and decoder of the generator, to improve the quantitative results regarding previous work and benchmark results. The proposed approach shows an improvement concerning previous work. It achieves better results on state-of-the-art literature approaches—the best approaches are from the second challenge on SR thermal images in terms of PSNR and SSIM quantitative measures. The current approach is trained using an unpaired set of images. The first dataset has large variability, showing that it is good enough for thermal image SR.

## Figures and Tables

**Table 1:** Average quantitative results on the evaluation set presented in Section 3.3. ${(}^{+})$ Best approaches at the PBVS-CVPR2021 challenge (Evaluation 2). ${(}^{1})$ Proposed approach trained with just the first dataset (without attention module); ${(}^{2})$ Proposed approach trained with both datasets (without attention module). ${(}^{3})$ Proposed approach trained with just first dataset and by using the proposed attention module. ${(}^{4})$ Proposed approach trained with both datasets and using the proposed attention module. Bold and underline values correspond to the first and second best results, respectively.

Approaches	PSNR	SSIM
Our Previous Work [16]	22.42	0.7989
NPU-MPI-LAB${}^{+}$ [18]	21.96	0.7618
SVNIT-NTNU-2${}^{+}$ [18]	21.44	0.7758
ULB-LISA${}^{+}$	22.32	0.7899
Current Work ${}^{1}$ (PA-D1)	22.98 (±2.02)	0.7991 (±0.0829)
Current Work ${}^{2}$ (PA-D1-D2)	21.93 (±2.07)	0.8117 (±0.0656)
Current Work ${}^{3}$ (PA-D1-AT)	**23.19 (±2.01)**	0.8023 (±0.0751)
Current Work ${}^{4}$ (PA-D1-D2-AT)	21.23 (±2.03)	**0.8167 (±0.0619)**

## Data Availability

https://github.com/rafariva/unsupervisedThSR (accessed on 12 December 2021).

## Abbreviations

SR	Super-Resolution
SISR	Single Image Super-Resolution
LR	Low-Resolution
MR	Mid-Resolution
HR	High-Resolution
GT	Ground Truth
ML	Machine Learning
CNN	Convolutional Neural Networks
LWIR	Long-Wavelength InfraRed
GAN	Generative Adversarial Network
AM	Attention Module
ROI	Region Of Interest
PA	Proposed Approach
PSRN	Peak Signal-to-Noise Ratio
SSIM	Structural Similarity Index Measure
